# Toxin-Antitoxin Systems Are Important for Niche-Specific Colonization and Stress Resistance of Uropathogenic *Escherichia coli*

**DOI:** 10.1371/journal.ppat.1002954

**Published:** 2012-10-04

**Authors:** J. Paul Norton, Matthew A. Mulvey

**Affiliations:** Division of Microbiology and Immunology, Pathology Department, University of Utah School of Medicine, Salt Lake City, Utah, United States of America; Institut Pasteur, France

## Abstract

Toxin-antitoxin (TA) systems are prevalent in many bacterial genomes and have been implicated in biofilm and persister cell formation, but the contribution of individual chromosomally encoded TA systems during bacterial pathogenesis is not well understood. Of the known TA systems encoded by *Escherichia coli*, only a subset is associated with strains of extraintestinal pathogenic *E. coli* (ExPEC). These pathogens colonize diverse niches and are a major cause of sepsis, meningitis, and urinary tract infections. Using a murine infection model, we show that two TA systems (YefM-YoeB and YbaJ-Hha) independently promote colonization of the bladder by the reference uropathogenic ExPEC isolate CFT073, while a third TA system comprised of the toxin PasT and the antitoxin PasI is critical to ExPEC survival within the kidneys. The PasTI TA system also enhances ExPEC persister cell formation in the presence of antibiotics and markedly increases pathogen resistance to nutrient limitation as well as oxidative and nitrosative stresses. On its own, low-level expression of PasT protects ExPEC from these stresses, whereas overexpression of PasT is toxic and causes bacterial stasis. PasT-induced stasis can be rescued by overexpression of PasI, indicating that PasTI is a bona fide TA system. By mutagenesis, we find that the stress resistance and toxic effects of PasT can be uncoupled and mapped to distinct domains. Toxicity was specifically linked to sequences within the N-terminus of PasT, a region that also promotes the development of persister cells. These results indicate discrete, multipurpose functions for a TA-associated toxin and demonstrate that individual TA systems can provide bacteria with pronounced fitness advantages dependent on toxin expression levels and the specific environmental niche occupied.

## Author Summary

Toxin-antitoxin (TA) systems are widespread among prokaryotes, including many important human pathogens. It has long been hypothesized that TA systems contribute to bacterial pathogenesis, but clear-cut phenotypes associated with any individual TA system have not been described. Using bioinformatics, we demonstrate that distinct subsets of TA systems are linked with a major group of bacterial pathogens known as Extraintestinal Pathogenic *E. coli* (ExPEC). These bacteria are responsible for the majority of urinary tract infections worldwide, and are major causes of sepsis and meningitis. Using murine infection models with a reference uropathogenic ExPEC isolate, we found that three of the ExPEC-associated TA systems act independently to promote bacterial survival and persistence within the host urinary tract. Furthermore, we show that the toxin protein associated with one of these TA systems increases ExPEC stress resistance and persistence in the face of antibiotics. This work demonstrates the functional importance of specific TA systems to ExPEC pathogenesis, highlighting their potential as therapeutic targets.

## Introduction

Toxin-antitoxin (TA) systems consist of stable toxic proteins that are held in check by co-expression of labile antitoxin molecules, the nature of which distinguishes three classes of TA systems [1], [2]. Antitoxins made by type I TA loci are small antisense RNAs that suppress translation of the toxin genes [3], while the antitoxins encoded by type II and type III TA loci are, respectively, proteins and small RNAs that complex with and inactivate their cognate protein toxins [2], [4]. When freed to act, toxins encoded by TA loci can disrupt diverse bacterial cell processes, including peptidoglycan synthesis, the polymerization of cytoskeletal components, ribosome assembly, tRNA availability, and mRNA stability [5], [6], [7], [8], [9]. TA systems were initially characterized as plasmid-encoded genes that serve as addiction molecules, promoting the heritable maintenance of extra-chromosomal DNA within a bacterial population [10], [11]. However, TA systems are not solely plasmid-associated, and have been found in abundance within bacterial chromosomes from diverse species. The functional relevance of individual chromosomally encoded TA systems to bacterial fitness within the environment is oftentimes ambiguous, and even less understood is the impact of these systems on bacterial pathogenesis [12], [13].

Environmental stresses have been shown to stimulate the transcription of multiple chromosomal TA loci, leading to the idea that these systems may enhance bacterial survival under adverse conditions [14], [15], [16]. Others suggest that chromosomal TA loci represent junk DNA or selfish genetic elements, or that they primarily function akin to plasmid-encoded addiction molecules, acting to stabilize genomic parasites such as conjugative transposons and prophages [17], [18]. A recent bioinformatics-based study supporting this hypothesis concluded that TA loci are selfish genetic elements that likely do not bestow any fitness advantage to the bacteria in which they reside [19]. In some cases this may be true, but there is mounting evidence that some chromosomal TA systems play larger roles in bacterial physiology and pathogenesis. Much of this evidence is circumstantial, based on expression analysis of TA systems in bacteria in the presence of various environmental stresses. For example, paralogous TA systems within the *Caulobacter crescentus* genome are differentially expressed in response to heavy metal-, heat-, and nitric oxide-induced stresses [14], while in *Mycobacterium tuberculosis* several TA systems are upregulated under hypoxic conditions or following bacterial entry into macrophages [16]. One of these, VapBC, was recently shown to regulate the balance of metabolic processes within mycobacteria [20].

Among strains of *E. coli* at least 17 type II chromosomal TA systems have been identified [2]. A large subset of these was recently shown to act redundantly *in vitro* to enhance survival of a K-12 *E. coli* strain in the presence of antibiotics by driving the formation of dormant, stress resistant cells known as persisters [9]. Type II TA systems may also promote biofilm formation by some K-12 strains [21], [22], [23], and can be differentially expressed in response to DNA damage and nutrient deprivation [15]. Considering these observations, we were interested in understanding possible links between chromosomal type II TA systems and the fitness and virulence potential of extraintestinal pathogenic *E. coli* (ExPEC). These pathogens are able to colonize diverse niches within humans and other host animals and are a major cause of bacteremia, sepsis, peritonitis, neonatal meningitis, and urinary tract infections (UTIs) [24]. Here we report that, as a group, ExPEC isolates encode a discrete repertoire of type II TA systems, three of which can individually impact the fitness and persistence of a reference ExPEC isolate *in vivo* within the urinary tract. In addition, the toxin PasT encoded by one of these TA systems is shown to possess dual, concentration-dependent activities that either enhance bacterial stress resistance and growth or, alternatively, promote bacterial stasis and persister cell formation.

## Results

### ExPEC Encode a Limited Number of Known Type II TA Systems

**Figure 1:**
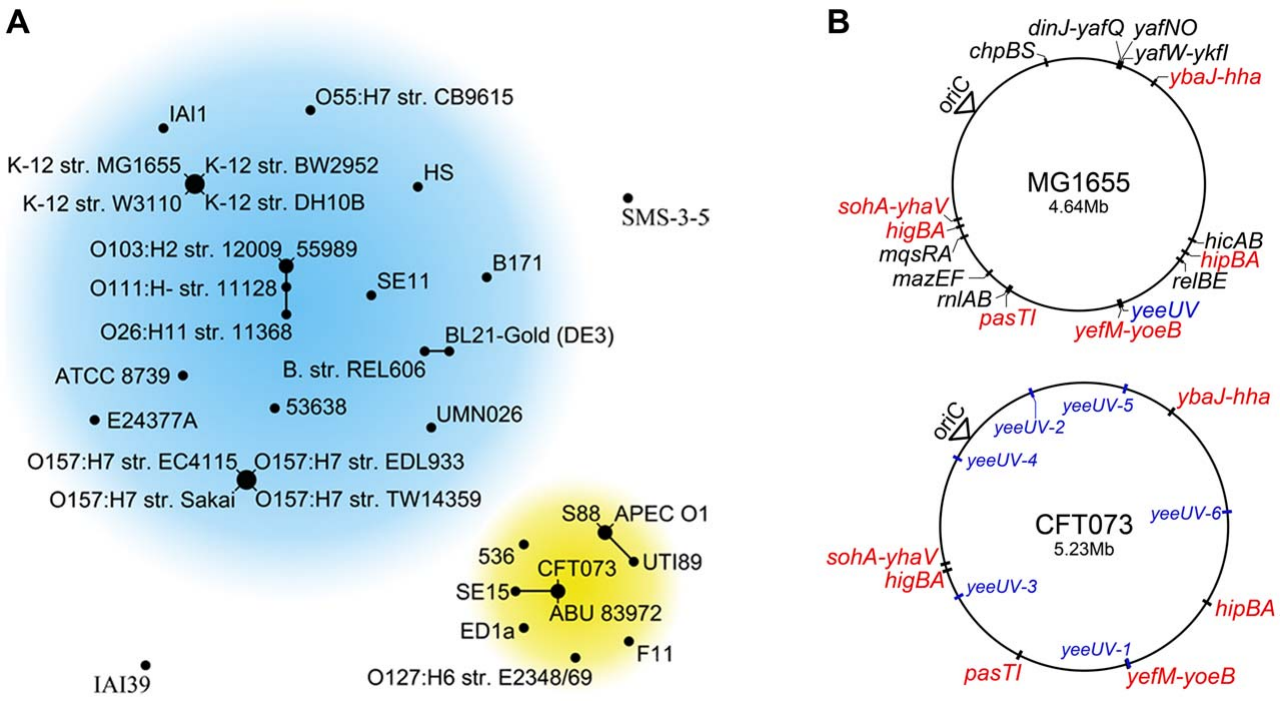
ExPEC encode distinct subsets of type II TA systems. (A) Based on similarities among 21 type II TA alleles, the eBURST algorithm clusters members of phylotype B2 (yellow) together, separate from other sequenced *E. coli* strains (blue). Strains connected by a line differ by a single TA allele. (B) Genomic maps denoting the relative locations of the type II TA systems encoded by MG1655 and CFT073. Red denotes TA systems that are present in both genomes; *yeeUV* and its allelic variants are shown in blue.

To ascertain potential patterns in the distribution and makeup of chromosomal type II TA systems among *E. coli* strains, the allelic profiles of 35 fully sequenced *E. coli* isolates were compared and organized based on similarities among 17 type II TA loci using the eBURST algorithm (Dataset S1) [25]. This allelic-based cluster analysis, which is often used to discern patterns of descent within bacterial populations in which horizontal gene transfer is widespread [19], identified two sizable groups of *E. coli* strains that differ in the number and composition of their type II TA systems (Figure 1A). One of these groups is comprised entirely of isolates belonging to the B2 phylotype, a specific subset of phylogenetically similar *E. coli* strains that includes most ExPEC isolates [26]. Interestingly, in our analysis the enteropathogenic *E. coli* (EPEC) strain E2348/69 was also grouped with the ExPEC isolates. This is, however, in line with a previous report that classified this EPEC strain as a member of the B2 phylotype [27]. These data indicate that, at least in the case of B2 strains, the composition of type II TA systems within *E. coli* isolates can reflect their phylogenetic origin, suggesting that specific TA systems may serve an evolutionarily conserved purpose within this cohort.

Relative to K-12 *E. coli* strains, we found that ExPEC generally have a reduced repertoire of intact type II TA loci, as exemplified by comparative analysis of the lab-adapted K-12 reference strain MG1655 and the ExPEC isolate CFT073 (Figure 1B). This pathogen was isolated from the blood of a patient with pyelonephritis and is part of a large sub-category of ExPEC known as uropathogenic *E. coli* (UPEC), the primary cause of UTIs worldwide [28]. Of the 16 type II TA loci encoded by MG1655, only seven are found in CFT073. The TA loci in CFT073 are syntenic with their counterparts in MG1655, although five additional, imperfect copies of one locus (*yeeUV*) are also present at sites around the CFT073 chromosome.

### TA Systems Provide Niche-Specific Benefits to ExPEC within the Host

**Figure 2:**
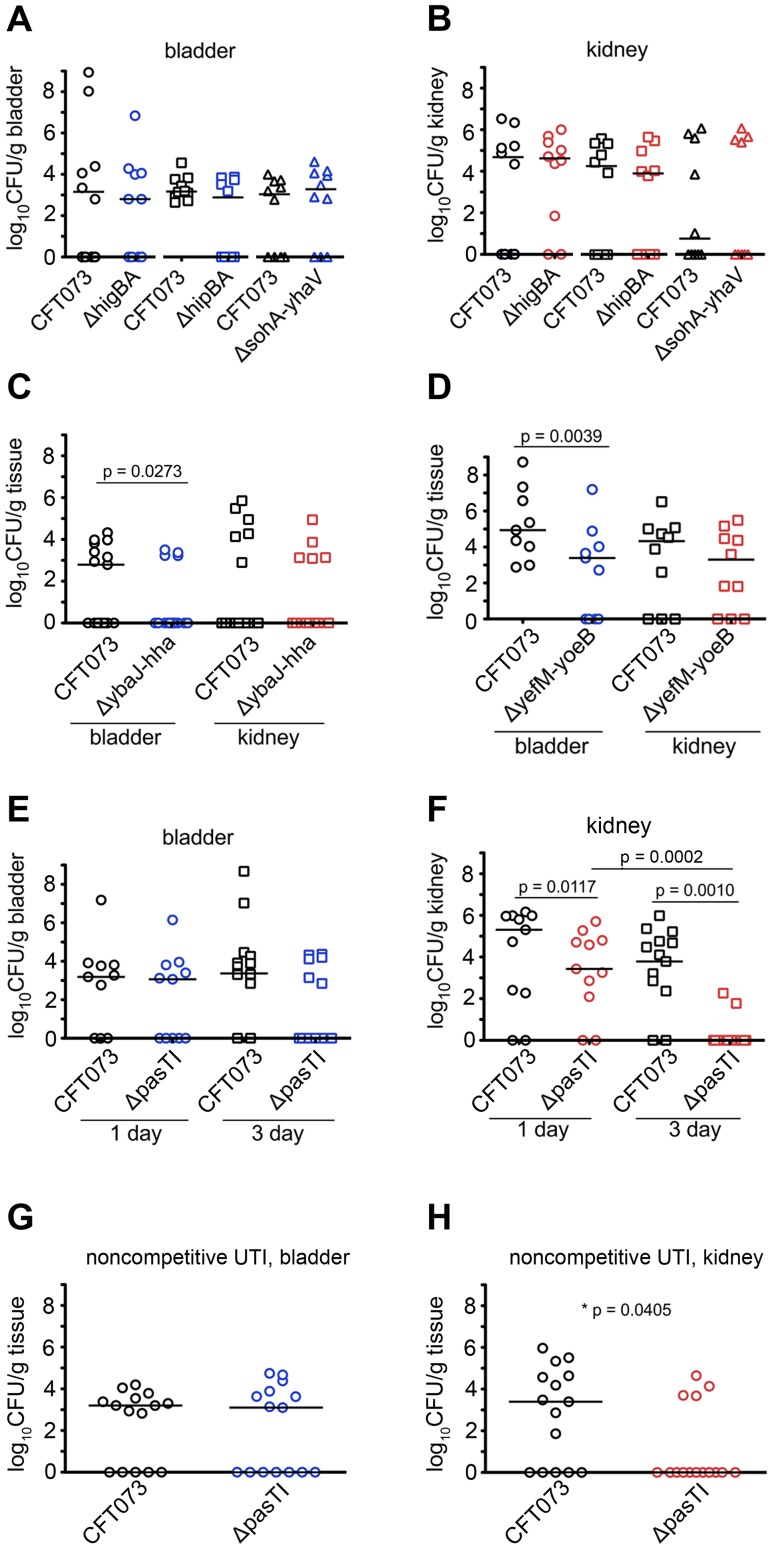
Distinct TA systems enhance UPEC fitness within the urinary tract. (A–H) Adult female CBA/J mice were infected via catheterization with equal numbers of wild type CFT073 and isogenic mutants lacking the indicated type II TA systems as part of either (A–F) competitive or (G and H) non-competitive assays. (A–H) Graphs show bacterial titers present in the bladder or kidneys, as indicated, at 3 d post-inoculation. (E and F) For competitive assays involving wild type CFT073 and CFT073Δ*pasTI*, bacterial titers recovered from mice at 1 d post-inoculation are also indicated. Bars denote median values for each group; n≥10 mice. *P* values determined using (A–F) Wilcoxon-matched paired signed rank and (G–H) Mann-Whitney U tests.

The relative conservation of the type II TA loci subsets among ExPEC isolates indicates that select TA systems may be important fitness determinants for these pathogens. To address this possibility, each single-copy type II TA locus in CFT073 was deleted and tested in competition with the wild type strain using a well-established mouse UTI model system [29], [30]. Cultures of wild type CFT073 and TA system mutant strains were mixed in a 1∶1 ratio and injected via transurethral catheterization into adult female CBA/J mice, and three days later bacterial titers in the bladder and kidneys were determined. In these assays, half of the six TA system mutants (Δ*higBA*, Δ*hipBA*, and Δ*sohA(prlF)-yhaV*) tested showed no significant defects relative to the wild type strain (Figure 2A and B). In contrast, mutants lacking either the *ybaJ-hha* (*tomB*-*hha*) or the *yefM-yoeB* TA locus were clearly outcompeted by wild type CFT073 in the bladder, but not the kidneys (Figure 2C and D), while the Δ*pasTI* mutant (CFT073Δ*pasTI*) was outcompeted only in the kidneys (Figure 2E and F). It should be noted that *pasTI* was originally dubbed *yfjGF* and then *ratAB*, but has been renamed here for reasons described later.

**Figure 3:**
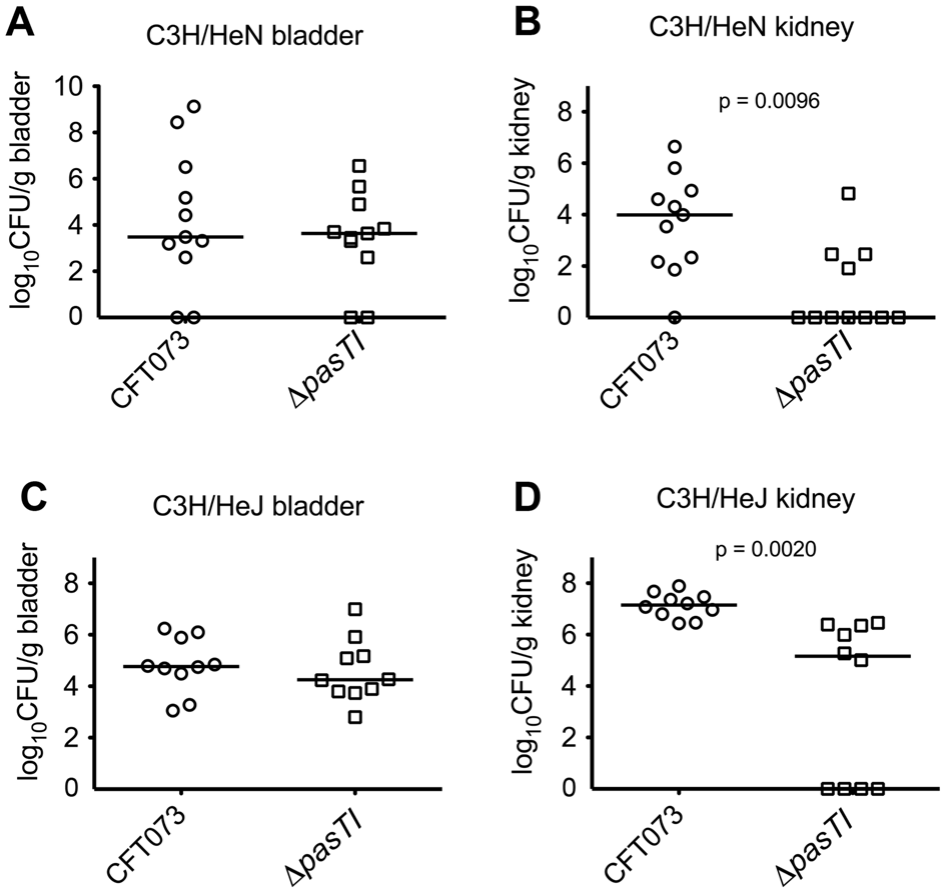
Defective colonization of the kidneys by CFT073Δ*pasTI* in C3H/HeN and C3H/HeJ mice. Adult female (A–B) C3H/HeN and (C–D) C3H/HeJ mice were infected via catheterization with equal numbers of wild type CFT073 and CFT073Δ*pasTI*. Graphs show bacterial titers present in the (A, C) bladders and (B, D) kidneys at 3 d post-inoculation. Bars indicate median values for each group; n≥10 mice per competitive assay. *P* values were determined using the Wilcoxon-matched paired signed rank test.

The competitive defect in kidney colonization by CFT073Δ*pasTI* was evident within 24 h post-inoculation and significantly worsened by the 3 d time point (Figure 2F). Over the same time frame, wild type CFT073 titers within the kidneys were not significantly diminished. We found that CFT073Δ*pasTI* is also significantly impaired in kidney colonization during non-competitive assays (Figure 2G and H). In contrast, defects observed with the Δ*ybaJ-hha* and Δ*yefM-yoeB* mutants in competitive assays (Figure 2C and D) were not manifest in non-competitive assays (Figure S1). The inability of CFT073Δ*pasTI* to effectively colonize the kidneys of CBA/J mice was recapitulated in experiments using two additional host strain backgrounds, C3H/HeN and C3H/HeJ (Figure 3). Of note, the Δ*pasTI* mutant was similar to wild type CFT073 in its ability to colonize and persist within the gastrointestinal tract of CBA/J mice (Fig. S2). Together, these results demonstrate that chromosomal type II TA systems like PasTI can provide significant advantages to UPEC within specific host environments.

### The PasTI TA System Promotes the Development of Persister Cells

**Figure 4:**
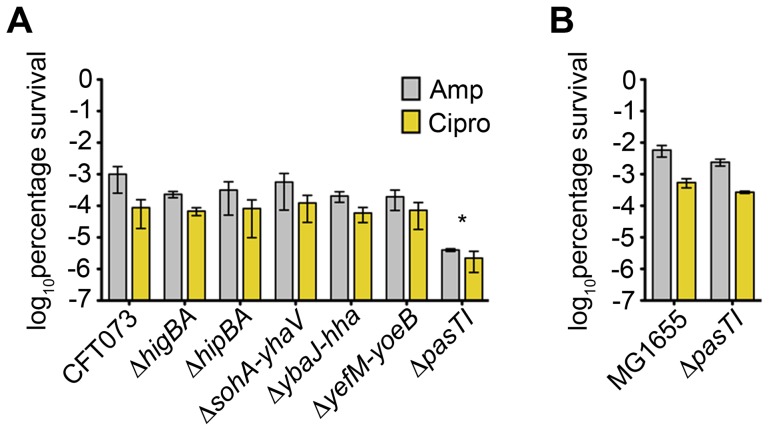
Persister cell formation by CFT073, but not MG1655, requires PasTI. Development of persister cells by (A) wild type CFT073 and associated type II TA system knockout mutants or (B) MG1655 and associated Δ*pasTI* mutant 5 h after the addition of 100 µg/mL ampicillin or 10 µg/mL ciprofloxacin to cultures in exponential growth phase. Data represent mean results ± SD from three independent experiments. *P* values were determined by Student's *t* test.

In K-12 *E. coli* strains, TA systems are proposed to act redundantly in the formation of persister cells in the presence of antibiotics and other harsh environmental stresses [31], [32]. For example, deletion of any one of the 10 mRNA endonuclease-encoding TA loci in the K-12 strain MG1655, individually, has no effect on bacterial persistence in the presence of either ampicillin or ciprofloxacin, whereas the successive deletion of five or more of these loci results in progressively decreased numbers of persisters [9]. In similar assays with CFT073 grown in LB broth, we found that deletion of the *pasTI* TA locus, alone, decreased bacterial persistence in the face of antibiotics by about 100-fold, while all other type II TA knockout mutants behaved like the wild type pathogen (Figure 4A). The minimal inhibitory concentrations of ampicillin and ciprofloxacin were the same for both wild type CFT073 and the Δ*pasTI* mutant (data not shown). MG1655, which has a larger pool of TA systems, was much more adept at forming persisters than CFT073, and the deletion of *pasTI* did not affect the ability of MG1655 to form persisters in our assays (Figure 4B). These data reveal that *pasTI* is dispensable for the development of persister cells by the K-12 strain, while in CFT073 the PasTI TA system acts in a more non-redundant fashion to promote persister cell formation. These results do not rule out the possibility that other gene products, including additional as-yet defined TA systems, may also contribute to the formation of persister cells by CFT073 and other UPEC isolates.

### PasT Enhances the Stress Resistance of UPEC

**Figure 5:**
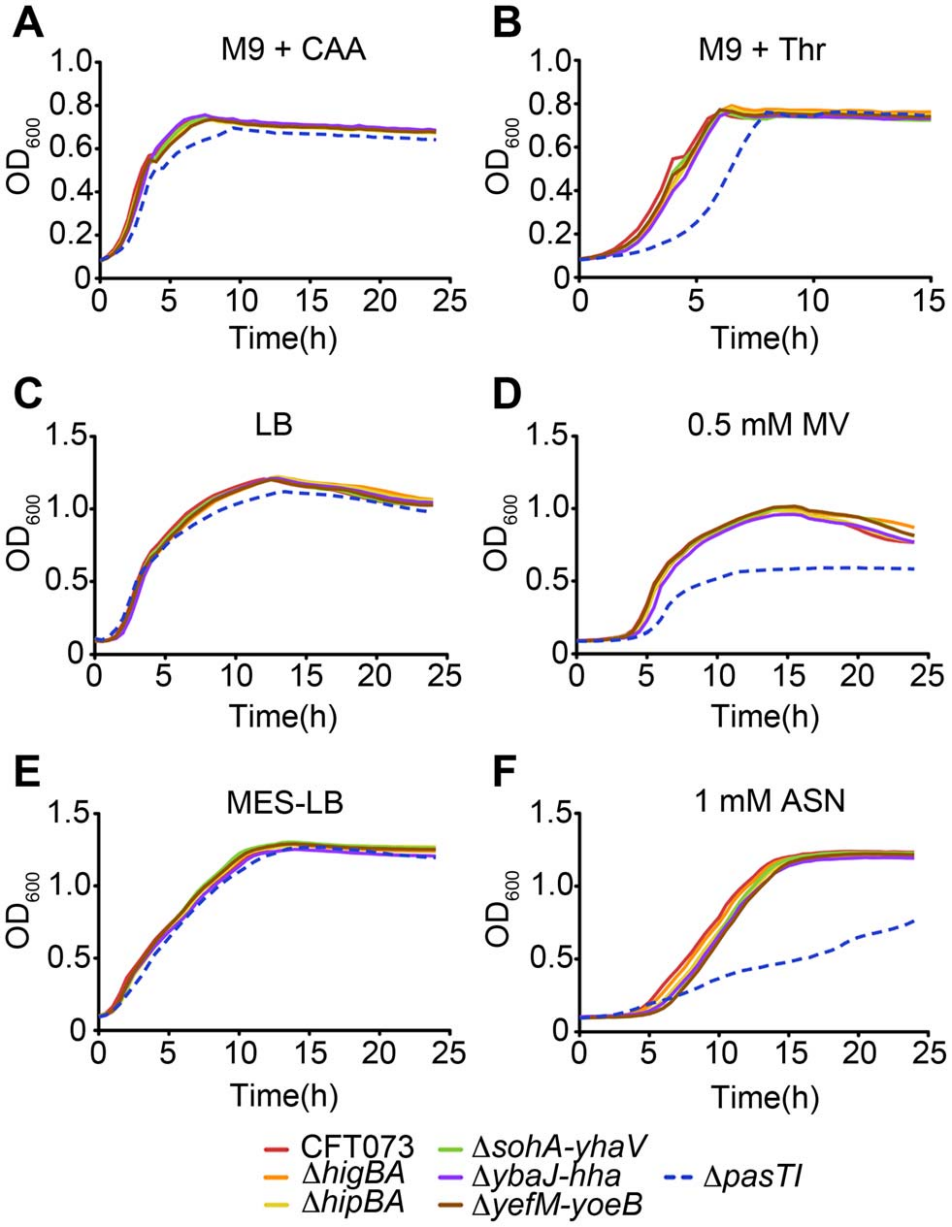
PasTI promotes the stress resistance of CFT073. Growth of wild type CFT073 and type II TA system knockouts in (A) M9 medium+0.2% casein amino acids, (B) a modified, lower-nutrient M9 medium+Thr, (C) LB broth, (D) LB broth containing 0.5 mM MV, (E) LB buffered to pH 5.0 with MES (MES-LB), and (F) MES-LB with 1 mM ASN. Graphs are representative of at least three independent experiments performed in quadruplicate.

**Figure 6:**
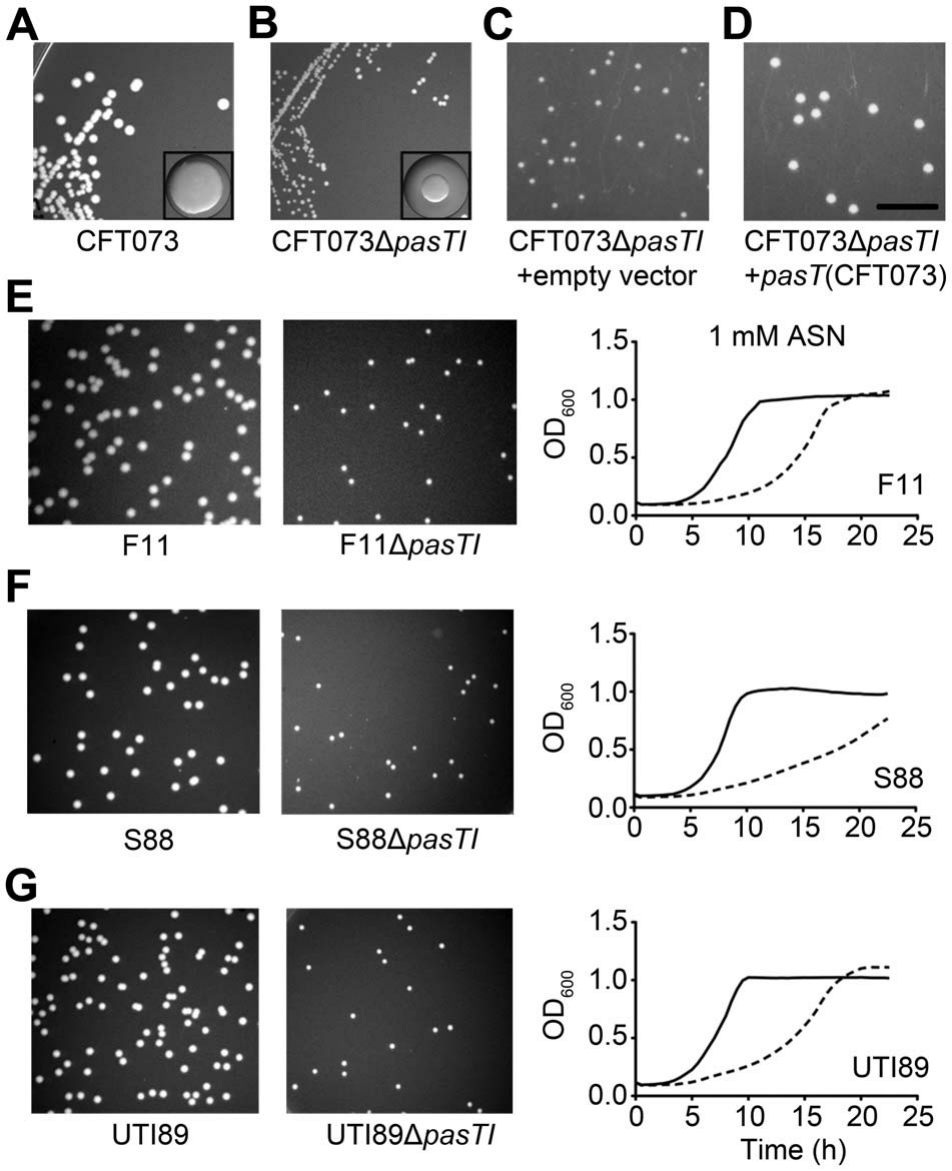
Deletion of *pasTI* reduces the stress resistance of multiple ExPEC isolates. (A–B) Images show wild type and CFT073Δ*pasTI* colonies on LB agar after overnight growth at 37°C; inset images show images of individual wild type and CFT073Δ*pasTI* colonies at identical magnification. (C–D) Colony sizes of CFT073Δ*pasTI* complemented with empty vector (pRR48) or pPN007, which allows for leaky expression of PasT from a P*tac* promoter. (E–G, left) Images show colonies of ExPEC strains and associated Δ*pasTI* mutants on LB agar after overnight growth at 37°C. (E–G, right) Growth of the ExPEC Δ*pasTI* mutants in 1 mM ASN is delayed compared to wild type. In each graph, solid lines represent the wild type strains and dashed lines indicate the Δ*pasTI* mutants. Images in A–G were taken at the same magnification (scale bar, 1 cm). All Δ*pasTI* colonies eventually reach wild type size.

During the course of a UTI, UPEC encounter multiple stresses, including nutrient deprivation and reactive nitrogen and oxygen species [30], [33], [34]. In standard LB broth and in M9 minimal medium supplemented with 0.2% casein amino acids, CFT073Δ*pasTI* grew like the wild type strain, but in M9 medium supplemented with only a single amino acid (40 µg/mL threonine), growth of the Δ*pasTI* mutant was significantly delayed (Figure 5A–C). CFT073Δ*pasTI* also displayed increased sensitivity to both oxidative and nitrosative stresses generated in broth cultures by addition of methyl viologen (MV) and acidified sodium nitrite (ASN), respectively (Figure 5D, F). Use of ASN in these assays involves the addition of sodium nitrite to MES-buffered LB broth (MES-LB, pH 5.0), leading to the generation of nitrous acid, NO, and other reactive nitrogen intermediates [35]. In control experiments, CFT073Δ*pasTI* grew normally in MES-LB without addition of ASN (Figure 5E). Corroborating these data, we observed on LB agar plates—which *E. coli* sense as a certifiable stress due in part to the presence of oxygen radicals [36]—that growth of CFT073Δ*pasTI* lags behind the wild type strain, resulting in a small colony phenotype (Figure 6A–B). Deletion of *pasTI* in other ExPEC isolates (including the neonatal meningitis isolate S88 and the UPEC strains F11 and UTI89) also resulted in small colony phenotypes as well as increased sensitivity to ASN (Figure 6E–G). None of the other type II TA system mutants in CFT073 were defective in these growth assays (Figure 5 and data not shown).

**Figure 7:**
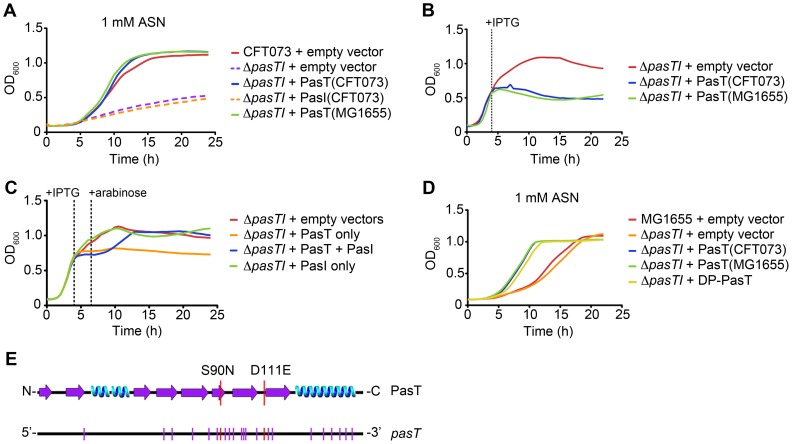
PasT has stress resistance and toxic effects, the latter of which can be countered by PasI. (A–C) Graphs show growth of recombinant CFT073 strains in (A) 1 mM ASN or (B, C) standard LB broth. (D) Curves show growth of recombinant MG1655 strains in 1 mM ASN. In B and C, IPTG and arabinose were added as indicated to induce high-level expression of PasT and PasI, respectively. (A, D) Alternatively, recombinant PasT and PasI were expressed at lower levels from a leaky P*tac* promoter. The *pasT* gene cloned from MG1655 was used as indicated. Otherwise, recombinant genes were derived from CFT073. Graphs are representative of at least 3 independent experiments performed in quadruplicate. (E) Bottom, the *pasT* gene carried by CFT073 and other ExPEC has a conserved set of synonymous (purple hashes) and two non-synonymous (red hashes) base pair changes relative to *pasT* encoded by MG1655 and many other *E. coli* strains. Top, the predicted secondary structure of PasT, with two conserved amino acid differences between the MG1655 and ExPEC-associated protein sequences highlighted.

The growth defects observed with CFT073Δ*pasTI* were attributable to loss of *pasT*, as low-level, leaky expression of PasT, but not PasI, from an un-induced P*tac* promoter complemented all growth defects observed with the Δ*pasTI* mutant (Figure 7A, Figure 6C–D, and data not shown). Leaky expression of PasT did not alter bacterial growth in standard LB broth (Figure S3A). Furthermore, expression of the entire *pasTI* operon under control of its native promoter complements the Δ*pasTI* mutant similar to expression of PasT alone, restoring wild type growth on LB agar plates and providing wild type levels of ASN resistance (Figure S3B and data not shown). PasT thus appears to enhance UPEC growth in the presence of diverse stresses, a phenomenon that is seemingly at odds with recent work showing that PasT (a.k.a. RatA) in K-12 *E. coli* acts as a toxin capable of binding 50S ribosomal subunits and thereby inhibiting translation and bacterial growth [7]. This discrepancy is partially reconciled by observations that high-level expression of PasT in CFT073 is toxic, resulting in growth arrest (Figure 7B). The induced expression of PasI counters the toxic effects of PasT, demonstrating that PasI can function as a *bona fide* antitoxin to PasT (Figure 7C). PasT is, therefore, conditionally toxic, depending in part on its expression levels and regulatory input from PasI.

### The Toxic and Stress Resistance Effects of PasT Are Separable

The *pasTI* locus is well conserved in all sequenced *E. coli* strains, with the exception of 23 nucleotide changes within the *pasT* gene that are found primarily in ExPEC isolates (Figure 7E). These ExPEC-associated alterations are silent, except for two that result in amino acid changes (S90N and D111E) relative to the K-12 sequence. Despite these differences, both K-12 and ExPEC versions of *pasT* enhanced the resistance of CFT073Δ*pasTI* to stresses like ASN, and both were similarly toxic when expressed at high levels (Figure 7A–B). Deletion of *pasTI* did not increase the sensitivity of MG1655 to ASN (Figure 7D), possibly due to input from other TA systems acting redundantly in this K-12 strain. However, low-level expression of *pasT* alleles did increase the resistance of MG1655 to ASN, indicating that the salubrious, non-toxic effects of PasT can be discerned in K-12 strains as well as pathogens under appropriate conditions.

**Figure 8:**
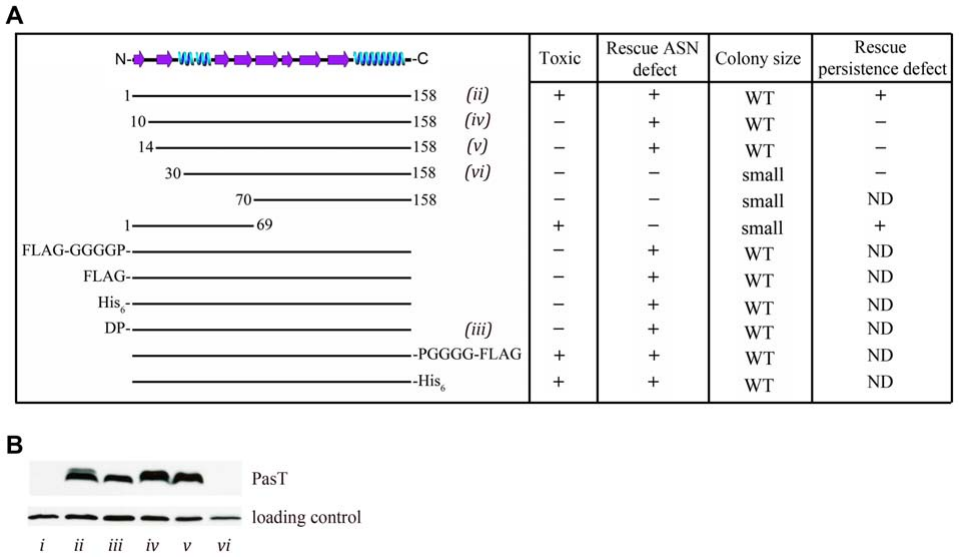
The toxic and salubrious effects of PasT can be genetically uncoupled. (A) Table indicates if the specified PasT truncation mutants and fusions display toxic, growth inhibitory effects when overexpressed in CFT073Δ*pasTI*, if their leaky expression rescues wild type (WT) growth of the Δ*pasTI* mutant in 1 mM ASN and on LB agar plates, and if their leaky expression rescues the ability of CFT073Δ*pasTI* to form persisters in the presence of ciprofloxacin (10 µg/mL). ND, not determined. (B) Western blot shows expression levels of C-terminal FLAG-tagged PasT constructs—denoted (ii)–(vi), as in (A)—following induction with IPTG. Lane (i) represents an empty vector control. A nonspecific band recognized by the anti-FLAG antibody was used as loading control. Equal loading was also verified by Coomassie staining (data not shown).

Using a series of genetic truncations and fusions, we next asked if the stress resistance and toxic, growth-inhibiting effects of PasT could be mapped to separable regions (Figure 8A). Removal of the N-terminal 9 to 13 amino acids of PasT completely abrogated its toxicity when overexpressed in CFT073Δ*pasTI*, as did fusion with N-terminal FLAG or His_6_ epitope tags or an even smaller two-amino acid (DP) addition. Although no longer toxic, these PasT variants were still able to restore wild type growth of CFT073Δ*pasTI* on agar plates and in the presence of ASN (Figure 8A). Overexpression of a non-toxic version of PasT (DP-PasT) also enhanced the resistance of MG1655 to nitrosative stress (Figure 7D). The addition of C-terminal epitope tags had no effect on PasT functionality (Figure 8A). Overexpression of just the N-terminal 69 residues of PasT was sufficient to induce growth arrest, but low-level expression of this PasT fragment provided no benefits on agar plates or upon exposure to ASN. As assessed by Western blot analysis, loss of PasT toxicity by modification of its N-terminus was not due to reduced expression of the non-toxic PasT variants relative to the full-length protein (Figure 8B). However, removal of the N-terminal 29 residues of PasT did render it barely detectable by western blot (Figure 8B), coordinate with loss of both the toxic and stress-resistance activities of this PasT mutant (Figure 8A). In total, these results demonstrate that the toxic and stress resistance effects of PasT can be uncoupled.

In light of these results, we next assessed the role of PasT in the development of persister cells by CFT073. The attenuated ability of CFT073Δ*pasTI* to form persisters in the presence of ciprofloxacin (Figure 4A), was rescued by low-level expression of either full-length PasT or the N-terminal 69 amino acid toxic domain (Figure 8A and Figure S4). In contrast, expression of non-toxic variants of PasT did not complement the Δ*pasTI* mutant in persister assays. We conclude that the same N-terminal toxic domain of PasT that halts bacterial growth when overexpressed also promotes the development of persister cells in the presence of antibiotic stress.

## Discussion

Chromosomal TA systems are widespread and functionally diverse, having the capacity to modulate an array of bacterial activities including phage resistance, biofilm formation, and persister cell development [17], [23], [37]. Results presented here extend these findings, demonstrating that type II TA systems can act independently within ExPEC to increase stress and antibiotic resistance as well as pathogen colonization and persistence within host tissues. Using a mouse UTI model we found that the YefM-YoeB and YbaJ-Hha TA systems each enhanced bladder colonization by the ExPEC isolate CFT073, but had no significant effect on bacterial colonization of the kidneys in competitive assays (Figure 2C–D). On the other hand, the PasTI TA system was not required for CFT073 survival within the bladder or intestinal tract but did promote pathogen colonization and persistence within the kidneys in both competitive and non-competitive assays (Figs. 2E–H, 3, and S2). These results indicate that individual TA systems can have profound effects on bacterial fitness within distinct host environments. It is feasible that phenotypes associated with some of the TA system mutants, but not discernable in the assays used in this study, may become evident by analysis of additional time points and possibly other hosts and environmental challenges.

The specific mechanisms by which these TA systems stimulate UPEC colonization of the host are likely complex. YoeB is an endoribonuclease that cleaves mRNA situated within the ribosomal A site [38], and the *yefM-yoeB* locus is one of 10 type II TA loci that can work cooperatively to facilitate persister cell formation by the K-12 *E. coli* strain MG1655 [9]. The toxin Hha acts differently, repressing the transcription of rare codon tRNAs and consequently inhibiting the expression of type 1 pili and the pore-forming toxin HlyA, among other genes [8], [39]. In K-12 *E. coli*, Hha activity can also stimulate bacterial cell lysis and biofilm dispersal [8]. In our assays, none of the type II TA system mutants, including Δ*yefM-yoeB* and Δ*ybaJ-hha*, were negatively affected in their ability to express type 1 pili or HlyA, and none were impaired in biofilm production in microtiter plate assays (data not shown). Furthermore, deletion of either *yefM-yoeB* or *ybaJ-hha* individually did not affect the development of persister cells in the face of ciprofloxacin or ampicillin (Figure 4A), leaving open the question of how these two TA systems contribute to UPEC survival within the bladder.

In contrast to the Δ*yefM-yoeB* and Δ*ybaJ-hha* strains, CFT073 lacking *pasTI* was significantly compromised in its capacity to form persister cells (Figure 4A). Our ability to detect clear phenotypic defects in these assays with CFT073Δ*pasTI*, but not with MG1655Δ*pasTI*, is likely attributable to decreased functional redundancy among the fewer TA systems carried by CFT073 relative to the K-12 strain [9]. The generation of persisters mediated by TA systems like PasTI may enhance the long-term survival of UPEC under hostile conditions within the host, including the administration of antibiotics used to treat UTIs [29]. However, persister cell formation does not appear to be the only way by which PasTI may promote UPEC survival *in vivo*. Specifically, low-level expression of PasT substantially increased the resistance of CFT073 to nitrosative and oxidative stresses, and enhanced growth of the pathogen under nutrient-limiting conditions. These environmental stresses are commonly encountered by UPEC during the course of a UTI [30], [40], [41], [42], [43]. Inherent differences between the bladder and kidney environments, such as the distribution of pathogen recognition receptors and dissimilar concentrations of various antimicrobial factors [44], [45], may account for the differential effects of *pasTI* deletion on bacterial fitness in the bladder versus the kidneys.

In this study, host defenses that may limit colonization of the kidneys by the Δ*pasTI* mutant were further assessed using C3H/HeJ mice (Figure 3). These animals have an impaired ability to respond to lipopolysaccharide (LPS) and increased susceptibility to both bladder and kidney infections [46], [47]. The increased sensitivity of C3H/HeJ mice to kidney infections has been linked by quantitative trait loci analysis to a site on chromosome 6, with possible input from loci on chromosomes 1, 4, and 9 [46]. Specific genes suggested to mediate host resistance to kidney infections include those encoding Toll-like receptor 5 (Tlr5) and the antimicrobial peptide cathelicidin. In our assays, we observed elevated levels of both wild type CFT073 and the Δ*pasTI* mutant in the bladders and kidneys of infected C3H/HeJ mice, relative to control immunocompetent C3H/HeN animals. However, kidney colonization by CFT073Δ*pasTI* was significantly attenuated in both types of mice, despite the overall increased sensitivity of the C3H/HeJ animals to UTI. These results indicate that the PasTI system promotes UPEC resistance to factors present in the kidneys of both the C3H/HeJ and C3H/HeN strains. Our *in vitro* assays (Figure 5) suggest that these defensive host factors may include amino acid limitation, superoxide radicals, and reactive nitrogen species.

The *pasT* locus is predicted to encode an oligoketide cyclase, while the *pasI* gene product is predicted to assume a ubiquitin-like β-grasp fold, a structural motif that is present in a wide variety of functionally distinct proteins [48], [49]. Previous work demonstrated that PasT could bind 50S ribosomal subunits and thereby inhibit protein translation [7]. In that study, PasT was referred to as RatA, for **R**ibosomal **a**ssociated **t**oxin A. We opted to avoid use of this name in the current study to reduce confusion with i) the *ratA* antitoxin encoded by *Bacillus subtilis* [50] and ii) the unrelated RatA and RatA-like proteins that are expressed by *Salmonella* species as well as many ExPEC isolates (e.g. NP_754911). Consequently, we refer to RatAB here as PasTI, for **P**ersistence **a**nd **s**tress-resistance **T**oxin and **I**mmunity proteins.

PasT functioned like a toxin and inhibited bacterial growth in our assays only when expressed at high levels, similar to toxins within other TA systems. The toxic effects of PasT were completely reversible by induced expression of PasI, confirming that these two proteins can function as a TA pair (Figure 7). The toxic effects of PasT were also ameliorated by modification of its N-terminus, either by the removal or addition of amino acids. Although no longer toxic when overexpressed, many of these PasT variants still maintained their salubrious functions, enhancing bacterial resistance to oxidative and nitrosative stresses. Loss of toxicity associated with these mutant proteins was not due to diminished PasT levels within the bacteria, but instead likely results from altered substrate recognition and/or activity. Regardless of the mode of action, these data show that the toxic and salubrious effects of PasT are separable. Consequently, it is feasible that the disparate functions of PasT may be differentially regulated in order to optimize bacterial stress resistance and persister cell phenotypes in response to changing environmental pressures. This may be especially important to the PasTI-dependent development of persister cells in the presence of antibiotics, a process that requires the N-terminal toxic domain of PasT.

Relative to other *E. coli* isolates, ExPEC and other members of the B2 phylotype encode a condensed set of the known type II TA systems. *E. coli* strains are traditionally grouped within the B2 phylotype based in part on the presence of distinct virulence factors, such as fimbrial and toxin genes like *papA*, *sfa*/*foc*, *hly*, and *cnf1* [51], [52]. It is remarkable that members of the B2 phylotype can also be distinguished from other *E. coli* isolates based solely on the makeup of their type II TA systems (see Figure 1A and Dataset S1). This suggests a common lineage among B2 strains in which a core set of TA systems has been selected. The ability to discern clear-cut phenotypes associated with the deletion of individual TA loci in ExPEC indicates that the fitness of these pathogens is likely more dependent on specific TA systems than K-12 strains like MG1655. These findings reveal the potential utility of ExPEC for defining the functional relevance of discrete TA systems, while also highlighting TA systems as compelling targets for therapeutic intervention. For example, compounds that selectively disrupt TA systems like PasTI may effectively attenuate the survival and growth of ExPEC within the host while having nominal effects on commensal *E. coli* strains that encode a larger, seemingly more redundant repertoire of TA systems.

## Materials and Methods

### Ethics Statement

Mice used in this study were handled in accordance with protocols approved by the Institutional Animal Care and Use Committee at the University of Utah (Protocol number 10-02014), following US federal guidelines indicated by the Office of Laboratory Animal Welfare (OLAW) and described in the Guide for the Care and Use of Laboratory Animals, 8th Edition.

### Cluster Analysis of Type II TA Loci in Sequenced *E. coli* Strains

Specific allelic values were assigned to indicate the presence, absence, or truncation of the known type II TA loci encoded by *E. coli* strains. These values were used to create an allelic profile for each fully sequenced *E. coli* isolate. The compiled allelic profiles were analyzed using the eBURST algorithm (http://eburst.mlst.net/) with 1,000 bootstrap iterations to organize strains into clusters within groups that share 17 out of 21 alleles [25]. See Dataset S1 (Excel) for further details on the assembly, grouping, and analysis of type II TA alleles by the eBURST algorithm.

### Genomic Localization of Type II TA Systems

Genomic locations of known type II TA systems encoded by MG1655 and CFT073 were obtained from NCBI and mapped onto circles that are proportional to the genome size of each strain. Origins of replication (OriC) were determined based on homology to “oriC” sequence: ATCTATTTATTTAGAGATCTGTTCTATTGTGATCTCTTATTAGGATCGCACTGCCCTGTGGATAACAAGGATCCGGCTTTTAAGATCAACAACCTGGAAAGGATCATTAACTGTGAATGATCGGTGATCCTGGACCGTATAAGCTGGGATCAGAATGAGGGGTTATACACAACTCAAAAACTGAACAACAGTTGTTCTTTGGATAACTACCGGTTGATCCAAGCTTCCTGA.

### Bacterial Strains and Plasmids

All bacterial strains and plasmids used in this study are listed in Tables S1 and S2. *E. coli* strains MG1655, CFT073, UTI89, F11, and S88 have been described previously [28], [53], [54], [55]. PasT and PasI expression constructs were made using standard molecular biology techniques employing the plasmids pRR48 and pBAD33 [56], [57]. Gene expression from the P*tac* promoter in the pRR48 backbone was induced by addition of 500 µM isopropyl-β-D-thiogalactopyranoside (IPTG), while gene expression from the pBAD33 promoter was induced using 0.2% L-arabinose. Primers and restriction sites used to construct all plasmids are indicated in Table S3, along with primers used to verify each clone by sequencing. Antibiotics (50 µg/mL kanamycin, 20 µg/mL chloramphenicol, or 100 g/mL ampicillin) were added to plates and growth medium to select for and maintain plasmids when necessary.

Targeted gene knockouts were generated in CFT073, MG1655, UTI89, F11, and S88 using the lambda Red-mediated linear transformation system [58], [59]. Briefly, a kanamycin resistance cassette was amplified from pKD4 with 40-base pair overhangs specific to the 5′ and 3′ ends of each target TA system locus (*higBA, hipBA, sohA-yhaV, ybaJ-hha, yefM-yoeB,* or *pasTI*). PCR products were introduced via electroporation into the strains carrying pKM208, which encodes for an IPTG-inducible lambda red recombinase. Knockouts were confirmed by PCR using the primers listed in Table S3. The chloramphenicol resistant cassette amplified from pKD3 was similarly added to the chromosome of CFT073, inserted within the intergenic region between genes *c3028* and *c3029* to create CFT073-Clm^R^. This strain serves as a tagged wild type control in the gastrointestinal tract colonization assays.

### Mouse UTI Model

Seven- to nine-week old female CBA/J mice (Jackson Labs), C3H/HeN mice (Harlan Laboratories), or C3H/HeJ mice (Jackson Labs) were used in accordance with IACUC-approved protocols as previously described [29], [60], [61]. Mice were anesthetized using isoflurane inhalation and inoculated via transurethral catheterization with 50 µL of a bacterial suspension containing approximately 1×10^7^ bacteria. CFT073 and isogenic knockout mutants were grown statically for 24 h in M9 medium, pelleted by spinning at 10,000 r.c.f. for 8 min, and resuspended in phosphate buffered saline (PBS) prior to inoculation. For competition assays, wild type and mutant strains were mixed 1∶1 prior to inoculation. The 1∶1 inoculation dosage was confirmed by plating serial dilutions on LB plates and LB plates containing 50 µg/mL kanamycin. Bladders and kidneys were recovered 1 or 3 d later, as indicated, and each was weighed and homogenized in 1 mL containing 0.025% Triton X-100. Homogenates were serially diluted and plated on LB agar plates to determine the number of bacteria per gram of tissue. For competition assays, plates containing kanamycin (50 µg/mL) were used to identify and enumerate the TA system knockout mutants, which carried Kan^R^ cassettes. Mouse experiments were repeated at least twice, and the total combined data from 10 or more animals is presented.

### UPEC Colonization of the Murine Gastrointestinal Tract

Cultures of CFT073-Clm^R^ (containing a chromosomal chloramphenicol-resistance cassette, *clm^R^*) and CFT073Δ*pasTI* (*kan^R^*) grown 20 mL static in M9 broth were pelleted and resuspended in PBS. Adult female CBA/J mice were each gavaged with 50 µL of the bacterial suspension containing 1×10^9^ CFU. Feces (∼100 mg) were collected at the indicated time points, weighed, and resuspended in 1 mL 0.7% NaCl. Serial dilutions were plated on LB agar containing either chloramphenicol (10 µg/mL) or kanamycin (50 µg/mL) in order to distinguish the Δ*pasTI* mutant and the Clm^R^-tagged strains. Mice receiving the CFT073 strains showed no overt signs of sickness. No chloramphenicol- or kanamycin-resistant bacteria were recovered in the feces of untreated mice.

### Statistics

Results from *in vivo* competition assays were analyzed by Wilcoxon matched-pairs signed rank test. Results from non-competitive assays in mice, including comparisons between 1- and 3-day titers of wild type CFT073 and CFT073Δ*pasTI* in the kidneys, were analyzed by Mann-Whitney two-tailed *t* tests. All statistical tests were performed using Prism 5.0c (GraphPad Software, Inc.). *P* values less than 0.05 are considered significant.

### Persister Assays

Persister assays were carried out with CFT073, MG1655, and their derivatives as previously described [9]. Briefly, 1 µg/mL ciprofloxacin or 100 µg/mL ampicillin was added to logarithmically growing bacterial cultures in LB broth and 5 h later the cultures were pelleted, washed once with PBS, and surviving bacteria were serially diluted and plated on LB agar. Total numbers of persister cells were calculated by dividing the number of viable bacteria present after antibiotic treatment by the number present prior to antibiotic addition.

### Growth Assays

Cultures of CFT073, MG1655 and their derivatives were grown shaking overnight at 37°C in 5 mL of LB broth, 100 mM morpholineethanesulfonic acid (MES)-buffered LB (MES-LB; pH 5.0), or modified M9 minimal medium+casein amino acids (6 g/L Na_2_HPO_4_, 3 g/L KH_2_PO_4_, 1 g/L NH_4_Cl, 0.5 g/L NaCl, 1 mM MgSO_4_, 0.1 mM CaCl_2_, 0.1% glucose, 0.0025% nicotinic acid, 16.5 µg/mL thiamine, and 0.2% casein amino acids). Overnight cultures were diluted 1∶100 and growth of quadruplicate 200-µl samples in 100-well honeycomb plates was assessed at 37°C using a Bioscreen C instrument (Growth Curves USA). Bacteria assayed for growth in 1 mM acidified sodium nitrite (ASN) were first grown from frozen stocks overnight in MES-LB. Strains assayed for growth in more nutrient-limited, M9 medium supplemented with threonine (6 g/L Na_2_HPO_4_, 3 g/L KH_2_PO_4_, 1 g/L NH_4_Cl, 0.5 g/L NaCl, 1 mM MgSO_4_, 0.1 mM CaCl_2_, 0.2% glucose, 0.0025% nicotinic acid, 40 µg/mL threonine, and 16.5 µg/mL thiamine) were initiated from cultures grown overnight in standard M9 medium. IPTG (500 µM) and 0.2% L-arabinose were added to cultures to induce high-level expression of PasT or PasI, as indicated. Methyl viologen (MV; a.k.a. paraquat) and ASN were prepared fresh prior to addition to LB or MES-LB broth cultures, respectively. All reagents were obtained from Sigma-Aldrich.

### Allelic Variants of *pasT* and Predicted Domain Structure of the Protein

The *pasT* nucleotide sequences of from ExPEC and K-12 strains were aligned and compared using ClustalX (downloaded at http://www.clustal.org/clustal2/) [62]. The secondary structure of PasT was predicted using Protein Homology/analogY Recognition Engine V 2.0 (Phyre^2^; http://www.sbg.bio.ic.ac.uk/phyre2/html/page.cgi?id=index) [49].

### Protein Analysis

Cultures of each strain indicated were grown overnight shaking in LB broth plus 100 µg/mL ampicillin (added for plasmid retention). Cultures were diluted 1∶50 into fresh LB broth and grown shaking at 37°C for 5 h, at which point IPTG was added to each culture to a final concentration of 500 µM. After an additional 1 h incubation, 1 mL of each culture was pelleted and resuspended in 200 µL SDS-TE (0.5% SDS in Tris-EDTA, pH 8.0). Proteins were resolved by SDS-PAGE and transferred to Immobilon PVDF-FL membrane (Millipore). Western blots were probed using ANTI-FLAG M2 antibody (Sigma-Aldrich) and visualized by enhanced chemiluminescence as previously described [63].

## Supporting Information

Dataset S1 **Tables used to define the Type II TA system allelic profiles of** ***E. coli*** **by eBURST analysis.** (Tab 1) Table showing TA system content among sequenced *E. coli*. (Tab 2) Table showing TA system content among phylotype B2 ExPEC strains. (Tab 3) Graph of the relative abundance of TA systems among all sequenced *E. coli* strains versus phylotype B2 isoaltes. (Tab 4) Table shows the individual allelic profiles of type II TA systems generated from Tab 1. (Tab 5) Table of allelic profiles that were used for input into the eBURST algorithm. (Tab 6) Separate tables with representative output from the eBURST algorithm, showing how the strains that are clustered together shift dependent upon based on the number of shared type II TA alleles considered.
(XLS)

Figure S1 **The CFT073Δ** ***yefM-yoeB*** **and CFT073Δ** ***ybaJ-hha*** **mutants colonize the murine urinary tract at similar levels to wild type CFT073 in noncompetitive assays.** (A–D) Adult female C3H/HeN and C3H/HeJ mice were infected via catheterization with 10^7^ CFU of wild type CFT073, CFT073Δ*yefM-yoeB* or CFT073Δ*ybaJ-hha*. Graphs show bacterial titers present in the (A) bladders and (B) kidneys at 3 d post-inoculation. Bars indicate median values for each group; n≥10 mice. *P* values were determined using Mann-Whitney U tests.
(TIF)

Figure S2 **CFT073 does not require** ***pasTI*** **for colonization of the murine gastrointestinal tract.** Adult female CBA/J mice were each gavaged with 50 µL of a bacterial suspension containing 1×10^9^ CFU CFT073-Clm^R^ or CFT073Δ*pasTI* (*kan^R^*). CFT073-Clm^R^ served as the wild type control in these assays. Gastrointestinal tract colonization was assessed by enumerating total CFU of the mutant and wild type strains per gram of feces collected at the indicated time points. Data represent mean CFU/g feces ± SEM. n = 3 to 5 mice.
(TIF)

Figure S3 **Low-level expression of PasT does not alter growth of the Δ** ***pasTI*** **mutant, while transcription of PasTI from its native promoter provides resistance to ASN.** Curves show growth of CFT073 and its derivatives in (A) LB broth and (B) MES-LB broth+1 mM ASN. (A) Leaky expression of *pasT* from a P*tac* promoter or expression of *pasTI* from its native promoter does not affect the growth of CFT073Δ*pasTI* in standard LB broth. In these assays, pRR48 served as an empty vector control. (B) Complementation of the Δ*pasTI* mutant growth defect in 1 mM ASN by the *pasTI* operon from its native promoter. Graphs are representative of at least two independent experiments performed in quadruplicate.
(TIF)

Figure S4 **Persister cell formation by CFT073 requires the toxic domain of PasT.** Graph shows numbers of viable bacteria (persisters) recovered 5 h after the addition of ciprofloxacin (10 µg/L) to broth cultures in exponential growth phase. The plasmid pRR48 serves as an empty vector control. Plasmid pPN007 encodes full-length PasT, pPN055 encodes the N-terminal 69 amino acids of PasT, and pPN068–069 encode non-toxic PasT variants lacking portions of the PasT N-terminus (see Figure 8). Data represent mean results ± SD from three independent experiments. *P* values were determined by Student's *t* test; ns indicates non-significant differences between the complemented strain and CFT073Δ*pasTI*/pRR48.
(TIF)

Table S1 **Strains used in this study.**
(PDF)

Table S2 **Plasmids used in this study.**
(PDF)

Table S3 **Primers used in this study.**
(PDF)
