# Supplementary figures and images for: Toxin-Antitoxin Systems Are Important for Niche-Specific Colonization and Stress Resistance of Uropathogenic Escherichia coli
Source: PLoS Pathog. 2012 Oct 4;8(10):e1002954. doi: 10.1371/journal.ppat.1002954 (PMC3464220; doi:10.1371/journal.ppat.1002954)

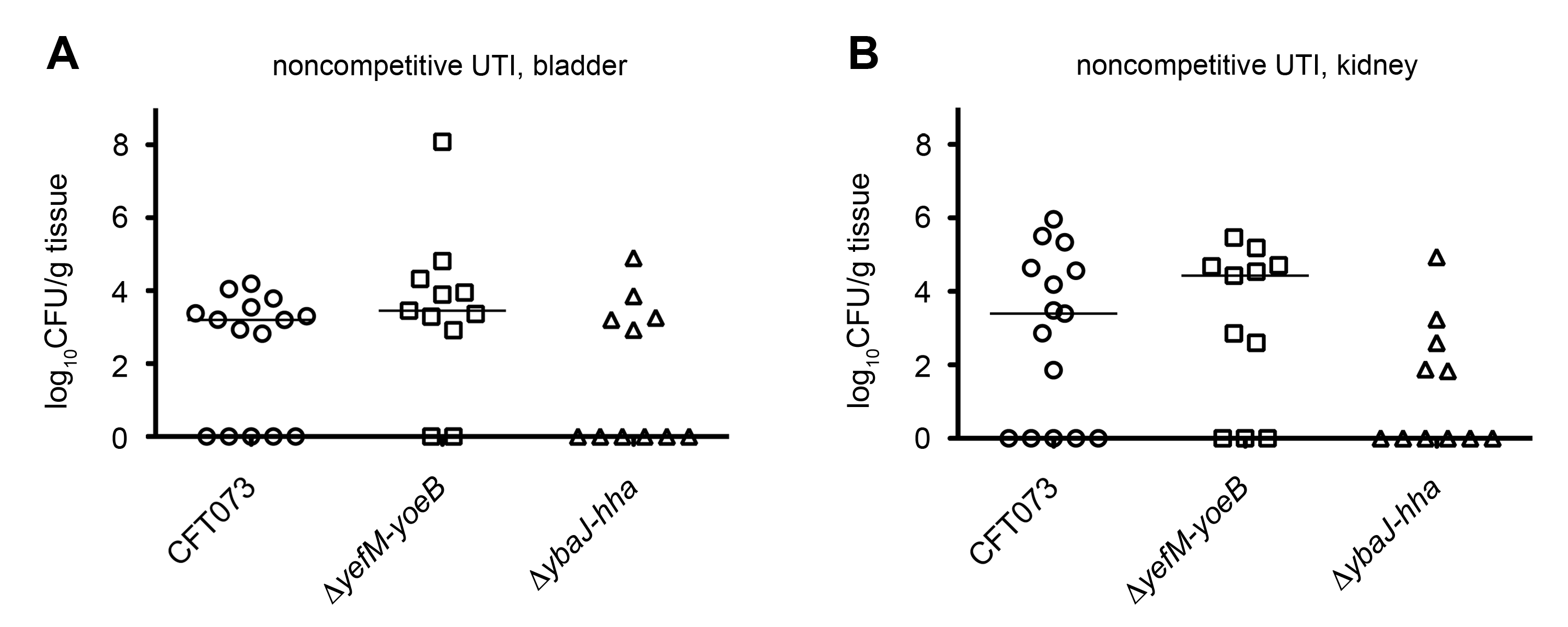

Supplement: Figure S1 — The CFT073Δ yefM-yoeB and CFT073Δ ybaJ-hha mutants colonize the murine urinary tract at similar levels to wild type CFT073 in noncompetitive assays. (A–D) Adult female C3H/HeN and C3H/HeJ mice were infected via catheterization with 107 CFU of wild type CFT073, CFT073ΔyefM-yoeB or CFT073ΔybaJ-hha. Graphs show bacterial titers present in the (A) bladders and (B) kidneys at 3 d post-inoculation. Bars indicate median values for each group; n≥10 mice. P values were determined using Mann-Whitney U tests. (TIF) [file ppat.1002954.s002.tif]

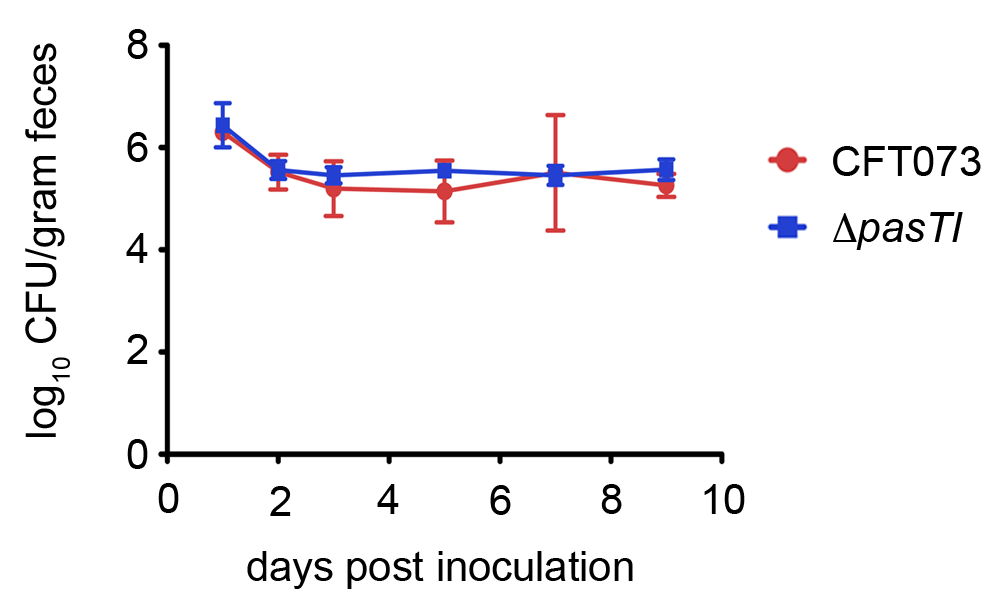

Supplement: Figure S2 — CFT073 does not require pasTI for colonization of the murine gastrointestinal tract. Adult female CBA/J mice were each gavaged with 50 µL of a bacterial suspension containing 1×10^9^ CFU CFT073-ClmR or CFT073ΔpasTI (kanR). CFT073-ClmR served as the wild type control in these assays. Gastrointestinal tract colonization was assessed by enumerating total CFU of the mutant and wild type strains per gram of feces collected at the indicated time points. Data represent mean CFU/g feces ± SEM. n = 3 to 5 mice. (TIF) [file ppat.1002954.s003.tif]

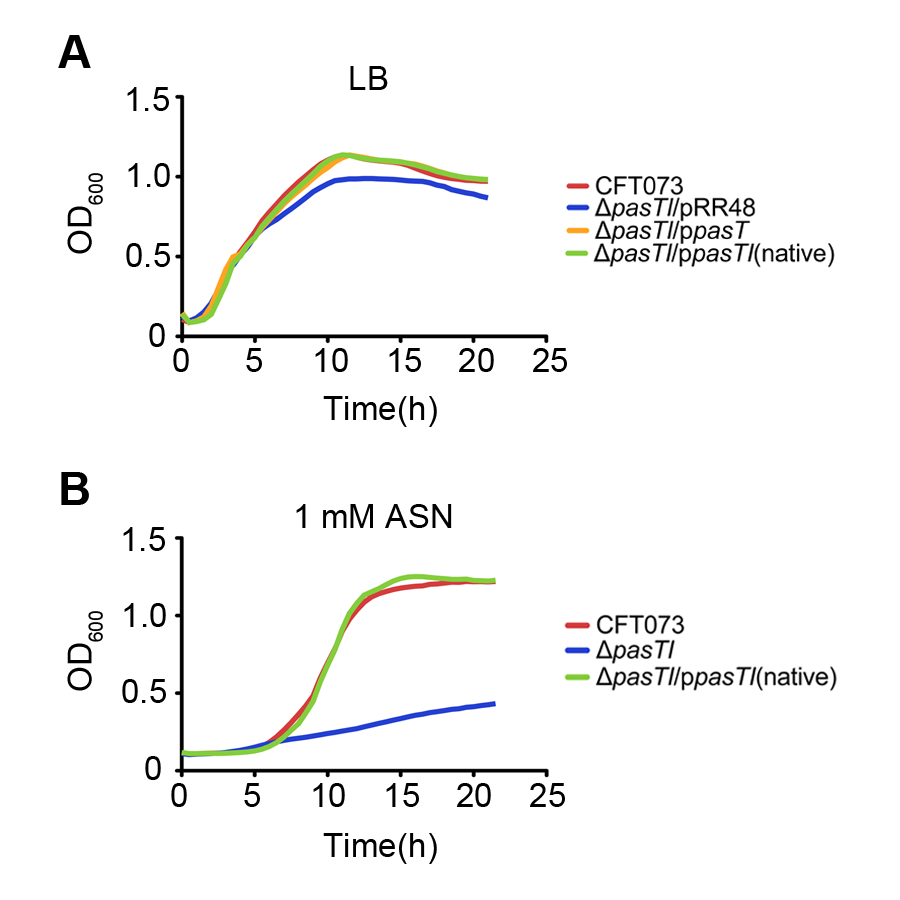

Supplement: Figure S3 — Low-level expression of PasT does not alter growth of the Δ pasTI mutant, while transcription of PasTI from its native promoter provides resistance to ASN. Curves show growth of CFT073 and its derivatives in (A) LB broth and (B) MES-LB broth+1 mM ASN. (A) Leaky expression of pasT from a Ptac promoter or expression of pasTI from its native promoter does not affect the growth of CFT073ΔpasTI in standard LB broth. In these assays, pRR48 served as an empty vector control. (B) Complementation of the ΔpasTI mutant growth defect in 1 mM ASN by the pasTI operon from its native promoter. Graphs are representative of at least two independent experiments performed in quadruplicate. (TIF) [file ppat.1002954.s004.tif]

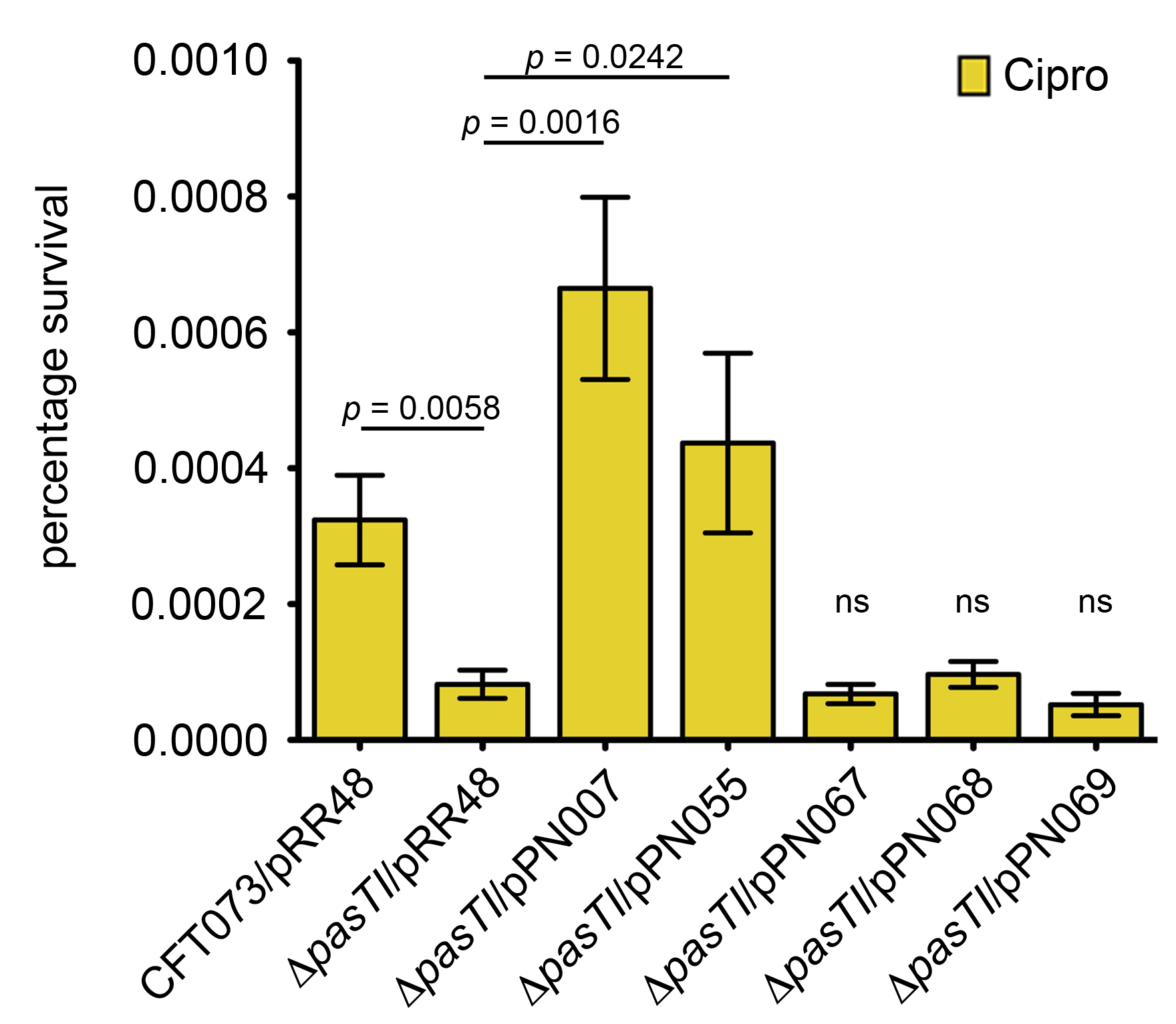

Supplement: Figure S4 — Persister cell formation by CFT073 requires the toxic domain of PasT. Graph shows numbers of viable bacteria (persisters) recovered 5 h after the addition of ciprofloxacin (10 µg/L) to broth cultures in exponential growth phase. The plasmid pRR48 serves as an empty vector control. Plasmid pPN007 encodes full-length PasT, pPN055 encodes the N-terminal 69 amino acids of PasT, and pPN068–069 encode non-toxic PasT variants lacking portions of the PasT N-terminus (see Figure 8). Data represent mean results ± SD from three independent experiments. P values were determined by Student's t test; ns indicates non-significant differences between the complemented strain and CFT073ΔpasTI/pRR48. (TIF) [file ppat.1002954.s005.tif]
