# Supplementary material for: Toxin-Antitoxin Systems Are Important for Niche-Specific Colonization and Stress Resistance of Uropathogenic Escherichia coli
Source: PLoS Pathog. 2012 Oct 4;8(10):e1002954. doi: 10.1371/journal.ppat.1002954 (PMC3464220; doi:10.1371/journal.ppat.1002954)
Supplement: Table S1 — Strains used in this study. (PDF) [file ppat.1002954.s006.pdf]

Table S1. Strains used in this study

Strain	Description	Source
MG1655	K-12 lab strain	[1]
CFT073	UPEC strain (pyelonephritis isolate, O6:K2:H1)	[2]
UTI89	UPEC strain (cystitis isolate, O18:K1:H7)	[3, 4]
F11	UPEC strain (O6:K2:H31)	[5]
S88	NMEC strain (O45:K1)	[6]
BW25141/pKD4	K-12 lab strain carrying plasmid pKD4	[7]
CFT073/pKM208	CFT073 carrying plasmid pKM208	This study
CFT073/pRR48	CFT073 carrying empty vector pRR48	This study
CFT073/pPN007	CFT073 carrying plasmid pPN007	This study
CFT073/pPN009	CFT073 carrying plasmid pPN009	This study
CFT073/pPN028	CFT073 carrying plasmid pPN028	This study
CFT073Δ*higBA*	CFT073 *higBA*::kan^R(pKD4)	This study
CFT073Δ*hipBA*	CFT073 *hipBA*::kan^R(pKD4)	This study
CFT073Δ*sohA-yhaV*	CFT073 *sohA-yhaV*::kan^R(pKD4)	This study
CFT073Δ*ybaJ-hha*	CFT073 *ybaJ-hha*::kan^R(pKD4)	This study
CFT073Δ*yefM-yoeB*	CFT073 *yefM-yoeB*::kan^R(pKD4)	This study
CFT073Δ*pasTI*	CFT073 *pasTI*::kan^R(pKD4)	This study
CFT073Δ*pasTI*/pRR48	CFT073Δ*pasTI* mutant carrying empty vector pRR48	This study
CFT073Δ*pasTI*/pBAD18-Cm/pRR48	CFT073Δ*pasTI* mutant carrying empty vectors pBAD18-Cm and pRR48	This study
CFT073Δ*pasTI*/pPN007	CFT073Δ*pasTI* mutant carrying plasmid pPN007	This study

CFT073Δ*pasTI*/pPN009	CFT073Δ*pasTI* mutant carrying plasmid pPN009	This study
CFT073Δ*pasTI*/pPN010	CFT073Δ*pasTI* mutant carrying plasmid pPN010	This study
CFT073Δ*pasTI*/pPN011	CFT073Δ*pasTI* mutant carrying plasmid pPN011	This study
CFT073Δ*pasTI*/pPN012	CFT073Δ*pasTI* mutant carrying plasmid pPN012	This study
CFT073Δ*pasTI*/pPN019	CFT073Δ*pasTI* mutant carrying plasmid pPN019	This study
CFT073Δ*pasTI*/pPN020	CFT073Δ*pasTI* mutant carrying plasmid pPN020	This study
CFT073Δ*pasTI*/pPN025	CFT073Δ*pasTI* mutant carrying plasmid pPN025	This study
CFT073Δ*pasTI*/pPN028	CFT073Δ*pasTI* mutant carrying plasmid pPN028	This study
CFT073Δ*pasTI*/pPN041	CFT073Δ*pasTI* mutant carrying plasmid pPN041	This study
CFT073Δ*pasTI*/pPN043	CFT073Δ*pasTI* mutant carrying plasmid pPN043	This study
CFT073Δ*pasTI*/pPN055	CFT073Δ*pasTI* mutant carrying plasmid pPN055	This study
CFT073Δ*pasTI*/pPN060	CFT073Δ*pasTI* mutant carrying plasmid pPN060	This study
CFT073Δ*pasTI*/pPN060/pPN007	CFT073Δ*pasTI* mutant carrying plasmid pPN060 and plasmid pPN007	This study
CFT073Δ*pasTI*/pPN064	CFT073Δ*pasTI* mutant carrying plasmid pPN064	This study
CFT073Δ*pasTI*/pPN067	CFT073Δ*pasTI* mutant carrying plasmid pPN067	This study
CFT073Δ*pasTI*/pPN068	CFT073Δ*pasTI* mutant carrying plasmid pPN068	This study
CFT073Δ*pasTI*/pPN069	CFT073Δ*pasTI* mutant carrying plasmid pPN069	This study
CFT073Δ*pasTI*/pPN078	CFT073Δ*pasTI* mutant carrying plasmid pPN078	This study
CFT073Δ*pasTI*/pPN079	CFT073Δ*pasTI* mutant carrying plasmid pPN079	This study
CFT073Δ*pasTI*/pPN080	CFT073Δ*pasTI* mutant carrying plasmid pPN080	This study
CFT073Δ*pasTI*/pPN081	CFT073Δ*pasTI* mutant carrying plasmid pPN081	This study

CFT073Δ*pasTI*/pPN083	CFT073Δ*pasTI* mutant carrying plasmid pPN081	This study
UTI89Δ*pasTI*	UTI89 *pasTI*::kan^R(pKD4)	This study
F11Δ*pasTI*	F11 *pasTI*::kan^R(pKD4)	This study
S88Δ*pasTI*	S88 *pasTI*::kan^R(pKD4)	This study
MG1655Δ*pasTI*	MG1655 *pasTI*::kan^R(pKD4)	This study
MG1655Δ*pasTI*/pRR48	MG1655Δ*pasTI* mutant carrying empty vector pRR48	This study
MG1655Δ*pasTI*/pPN007	MG1655Δ*pasTI* mutant carrying plasmid pPN007	This study
MG1655Δ*pasTI*/pPN009	MG1655Δ*pasTI* mutant carrying plasmid pPN009	This study
MG1655Δ*pasTI*/pPN028	MG1655Δ*pasTI* mutant carrying plasmid pPN028	This study
CFT073-Clm^R	CFT073 with a chloramphenicol resistance cassette inserted into the intergenic region of genes *c3028* and *c3029*	This study

Supplemental References

1. Blattner FR, Plunkett G, 3rd, Bloch CA, Perna NT, Burland V, et al. (1997) The complete genome sequence of *Escherichia coli* K-12. *Science* 277: 1453-1474.

2. Welch RA, Burland V, Plunkett G, 3rd, Redford P, Roesch P, et al. (2002) Extensive mosaic structure revealed by the complete genome sequence of uropathogenic *Escherichia coli*. *Proc Natl Acad Sci U S A* 99: 17020-17024.

3. Chen SL, Hung CS, Xu J, Reigstad CS, Magrini V, et al. (2006) Identification of genes subject to positive selection in uropathogenic strains of *Escherichia coli*: a comparative genomics approach. *Proc Natl Acad Sci U S A* 103: 5977-5982.

4. Mulvey MA, Schilling JD, Hultgren SJ (2001) Establishment of a persistent *Escherichia coli* reservoir during the acute phase of a bladder infection. *Infect Immun* 69: 4572-4579.

5. Lloyd AL, Rasko DA, Mobley HL (2007) Defining genomic islands and uropathogen-specific genes in uropathogenic *Escherichia coli*. *J Bacteriol* 189: 3532-3546.

6. Touchon M, Hoede C, Tenaillon O, Barbe V, Baeriswyl S, et al. (2009) Organised genome dynamics in the *Escherichia coli* species results in highly diverse adaptive paths. *PLoS Genet* 5: e1000344.

7. Datsenko KA, Wanner BL (2000) One-step inactivation of chromosomal genes in *Escherichia coli* K-12 using PCR products. *Proc Natl Acad Sci U S A* 97: 6640-6645.
