# Supplementary material for: Toxin-Antitoxin Systems Are Important for Niche-Specific Colonization and Stress Resistance of Uropathogenic Escherichia coli
Source: PLoS Pathog. 2012 Oct 4;8(10):e1002954. doi: 10.1371/journal.ppat.1002954 (PMC3464220; doi:10.1371/journal.ppat.1002954)
Supplement: Table S2 — Plasmids used in this study. (PDF) [file ppat.1002954.s007.pdf]

Table S2. Plasmids used in this study

Plasmid	Description	Source
pKD3	template plasmid for gene disruption; contains FRT flanked Clm^R cassette	[1]
pKD4	template plasmid for gene disruption; contains FRT flanked Kan^R cassette	[1]
pKM208	Amp^R; encodes IPTG-inducible lambda red recombinase	[2]
pRR48	Amp^R cloning plasmid containing an IPTG inducible P*tac* promoter	[3]
pBAD33	Clm^R cloning plasmid containing arabinose inducible pBAD promoter	[4]
pGEN-MCS	High retention Amp^R cloning plasmid	[5]
pPN007	Amp^R; *pasT* (from CFT073) cloned into PstI, HindIII of pRR48	This study
pPN009	Amp^R; *pasT* (from CFT073) cloned into BamHI, KpnI of pRR48	This study
pPN010	Amp^R; *pasI* (from CFT073) cloned into BamHI, KpnI of pRR48	This study
pPN011	Amp^R; *pasTI* (from CFT073) cloned into BamHI, KpnI of pRR48	This study
pPN012	Amp^R; *pasT* (from CFT073) with C-terminal FLAG cloned into PstI,	This study
pPN019	Amp^R; *pasT* (from CFT073) with GGGGP linker and C-terminal FLAG	This study
pPN020	Amp^R; *pasT* (from CFT073) with GGGGP linker and N-terminal FLAG	This study
pPN025	Amp^R; *pasT* (from CFT073) with N-terminal FLAG cloned into PstI,	This study
pPN028	Amp^R; *pasT* (from MG1655) cloned into PstI, HindIII of pRR48	This study
pPN041	Amp^R; *pasT* (from CFT073) with N-terminal 6X-His cloned into PstI,	This study
pPN043	Amp^R; *pasT* (from CFT073) with C-terminal 6X-His cloned into PstI,	This study
pPN055	Amp^R; first 207 nucleotides of *pasT* (from CFT073) cloned into PstI,	This study
pPN060	Clm^R; *pasI* (from CFT073) cloned into SacI, HindIII of pBAD33 with	This study
pPN064	Amp^R; nucleotides 208-477 of *pasT* (from CFT073) cloned into PstI,	This study

pPN067	Amp^R; nucleotides 28-477 of *pasT* (from CFT073) cloned into PstI,	This study
pPN068	Amp^R; nucleotides 40-477 of *pasT* (from CFT073) cloned into PstI,	This study
pPN069	Amp^R; nucleotides 88-477 of *pasT* (from CFT073) cloned into PstI,	This study
pPN078	Amp^R; *pasT* (from CFT073) with C-terminal FLAG tag cloned into	This study
pPN079	Amp^R; nucleotides 28-477 of *pasT* (from CFT073) with C-terminal	This study
pPN080	Amp^R; nucleotides 40-477 of *pasT* (from CFT073) with C-terminal	This study
pPN081	Amp^R; nucleotides 88-477 of *pasT* (from CFT073) with C-terminal	This study
pPN083	Amp^R; *pasTI* operon including native promoter (243 bp upstream of *pasTI*; all sequences from CFT073) cloned into HindIII, NcoI sites of pGEN-MCS	This study

Supplemental References

1. Datsenko KA, Wanner BL (2000) One-step inactivation of chromosomal genes in *Escherichia coli* K-12 using PCR products. *Proc Natl Acad Sci U S A* 97: 6640-6645.
2. Murphy KC, Campellone KG (2003) Lambda Red-mediated recombinogenic engineering of enterohemorrhagic and enteropathogenic *E. coli*. *BMC Mol Biol* 4: 11.
3. Zhou Q, Ames P, Parkinson JS (2009) Mutational analyses of HAMP helices suggest a dynamic bundle model of input-output signalling in chemoreceptors. *Mol Microbiol* 73: 801-814.
4. Guzman LM, Belin D, Carson MJ, Beckwith J (1995) Tight regulation, modulation, and high-level expression by vectors containing the arabinose PBAD promoter. *J Bacteriol* 177: 4121-4130.
5. Lane MC, Alteri CJ, Smith SN, Mobley HL (2007) Expression of flagella is coincident with uropathogenic *Escherichia coli* ascension to the upper urinary tract. *Proc Natl Acad Sci U S A* 104: 16669-16674.
