# Supplementary material for: Toxin-Antitoxin Systems Are Important for Niche-Specific Colonization and Stress Resistance of Uropathogenic Escherichia coli
Source: PLoS Pathog. 2012 Oct 4;8(10):e1002954. doi: 10.1371/journal.ppat.1002954 (PMC3464220; doi:10.1371/journal.ppat.1002954)
Supplement: Table S3 — Primers used in this study. (PDF) [file ppat.1002954.s008.pdf]

Table S3. Primers used in this study

Primer use/name		5'-3' Sequence
higBA KO		
	JPN150	**TGCTACATACGTGAAGTTATGACGCATAAAGAATACGATGTGTAGGCTGGAGCTGCTTCG**
	JPN151	**TTAATCAATAAACAAGGCGGGAGAAATGCCGAATCGCGTTCATATGAATATCCTCCTTAG**
higBA KO confirm		
	JPN152	**GCAATGTCTGCCCAGTTCTG**
	JPN153	**GCTGCGATGTAGCTATGGAG**
hipBA KO		
	JPN073	**TTGCTGTGGACGTATGACATGATGAGCTTTCAGAAGATTGTGTAGGCTGGAGCTGCTTCG**
	JPN074	**TTATGCGCACCAACATAAACCAAGGGAGAATCCAGTCGTTCATATGAATATCCTCCTTAG**
hipBA KO confirm		
	JPN075	**CAGCGTTTATCCCGTAGAG**
	JPN076	**GGAAGTCAGAAATCACAACG**
sohA-yhaV KO		
	JPN077	**ATGCCCGCTAATGCTCGCTCTAACGCTGTACTGACCACTGTGTAGGCTGGAGCTGCTTCG**
	JPN078	**TCAATGGCTTTCTTCTGTCTCTTGGGTGAGGCTTTCCCAGCATATGAATATCCTCCTTAG**
sohA-yhaV KO confirm		
	JPN079	**AGTCCACACATCCATTA**
	JPN080	**GTTGAAGTGATTCTGGTC**
ybaJ-hha KO		
	JPN154	**ATGGATGAATACTCACCCAAAAGACATGATATCGCACATGTGTAGGCTGGAGCTGCTTCG**
	JPN155	**TTAGCGAATAAATTTCCATACTGAGGAAGGGATCTTGTCGCATATGAATATCCTCCTTAG**

ybaJ-hha KO confirm

JPN156 **GCAAGTCCAGGTCAGTAAG**

JPN157 **CTGTGCTCATTACTGACTTC**

yefM-yoeB KO

JPN065 **ATGCGTACAATTAGCTACAGCGAAGCGCGTCAGAATTTTGTGTAGGCTGGAGCTGCTTCG**

JPN070 **TCAATAATGATAACGACATGCTGCAATGAGCAGTGAATCGCATATGAATATCCTCCTTAG**

yefM-yoeB KO confirm

JPN067 **CGTCTCGCTGACAATGTCTTG**

JPN072 **TTCATCCGTTATCTCCTG**

pasTI KO

JPN081 **ATGATATTATTTGTTGGATTTTTGTTGATGGAAATTGTTGTGTAGGCTGGAGCTGCTTCG**

JPN082 **TTATTTATTCGCTGATTTTTCTGCTCGTTGCCTGCGAAGCCATATGAATATCCTCCTTAG**

pasTI KO confirm

JPN083 **CTTAGCATGAACCCGATG**

JPN084 **GTTGCTCTCAAAGACGTT**

Clm^R KI

TW1 **TCTGGCGTAGCCTGGGAGTTATTGCCGGATGCGATGCTGGTGTGTAGGCTGGAGCTGCTTCG**

TW2 **TCACGTAAAAAAACGTCTAATCCGTAGACCGGATAAGAGGCATATGAATATCCTCCTTAG**

Clm^R KI confirm

TW3 **GGATGGACGAAGGTACTGGA**

TW4 **GGTGAGACACTGACCACACG**

pPN007

JPN212 **GCCCA CTGCAG ATGATATTATTTGTTGGAT**

JPN201 **TTCGG AAGCTT TTACCTGGCACTGTAGACCT**

pPN009

	JPN106	**GCCGC GGATCC G ATGATATTATTTGTTGGATT**
	JPN107	**ATTCG GGTACC TTACCTGGCACTGTAGACCT**

pPN010

	JPN108	**ATTCG GGATCC G GTGCCAGGTAAAATTGCCGT**
	JPN109	**CCCGC GGTACC TTATTTATTCGCTGATTTTT**

pPN011

	JPN106	**GCCGC GGATCC G ATGATATTATTTGTTGGATT**
	JPN109	**CCCGC GGTACC TTATTTATTCGCTGATTTTT**

pPN012

	JPN126	**CCCGG CTGCAG ATGATATTATTTGTTGGATT**
	JPN127	**ATCCG AAGCTT CTATCCCTTATCGTCGTCATCCTTGTAGTC CCTGGCACTGTAGACCTCT**

pPN019

	JPN126	**CCCGG CTGCAG ATGATATTATTTGTTGGATT**
	JPN141	**ATCCG AAGCTT CTATCCCTTATCGTCGTCATCCTTGTAGTC TGGTCCTCCTCCTCC CCTGGCACTGTAGACCTCT**

pPN020

	JPN140	**CCCGG CTGCAG GACTACAAGGATGACGACGATAAGGGA GGAGGAGGAGGACCA ATGATATTATTTGTTGGAT**
	JPN107	**ATTCG GGTACC TTACCTGGCACTGTAGACCT**

pPN025

	JPN160	**CCCGG CTGCAG GACTACAAGGATGACGACGATAAGGGA ATGATATTATTTGTTGGAT**
	JPN107	**ATTCG GGTACC TTACCTGGCACTGTAGACCT**

pPN028

	JPN183	**GACGG CTGCAG ATGATATTATTTGTTGGAT**
	JPN184	**TTCGG AAGCTT TTACCTGGCACTGTAAACC**

pPN041

JPN206	**ATCCGCTGCAGATGCACCACCACCACCACCACGGAATGATATTATTTGTTGGAT**
JPN207	**ATCCGAAGCTTTTACCTGGCACTGTAGACCT**

pPN043

JPN183	**GACGG CTGCAG ATGATATTATTTGTTGGAT**
JPN210	**ATCCG AAGCTT TTAGTGGTGGTGGTGGTGGTG CCTGGCACTGTAGACCTCTT**

pPN054

JPN211	**CGTCC GAGCTC ATGATATTATTTGTTGGAT**
JPN207	**ATCCGAAGCTTTTACCTGGCACTGTAGACCT**

pPN055

JPN212	**GCCCA CTGCAG ATGATATTATTTGTTGGAT**
JPN223	**CAGGT AAGCTT TTA AGCCTTAGACACATCTACCG**

pPN064

JPN232	**CACAG CTGCAG GGGATCAGCAAAACGTTTAC**
JPN233	**ATCCG AAGCTT TTACCTGGCACTGTAGACC**

pPN067

JPN236	**CTGAG CTGCAG ATGGAAATTGTTATGCCTC**
JPN201	**TTCGG AAGCTT TTACCTGGCACTGTAGACCT**

pPN068

JPN237	**CACAG CTGCAG ATGCCTCAGATTAGTCGGA**
JPN201	**TTCGG AAGCTT TTACCTGGCACTGTAGACCT**

pPN069

JPN239	**CGTAG CTGCAG ATGTATCAGTTAGTGAATG**
JPN201	**TTCGG AAGCTT TTACCTGGCACTGTAGACCT**

pPN078

JPN106 **GCCGC GGATCC G ATGATATTATTTGTTGGATT**

JPN127 **ATCCG AAGCTT CTATCCCTTATCGTCGTCATCCTTGTAGTC CCTGGCACTGTAGACCTCT**

pPN079

JPN236 **CTGAG CTGCAG ATGGAAATTGTTATGCCTC**

JPN127 **ATCCG AAGCTT CTATCCCTTATCGTCGTCATCCTTGTAGTC CCTGGCACTGTAGACCTCT**

pPN080

JPN237 **CACAG CTGCAG ATGCCTCAGATTAGTCGGA**

JPN127 **ATCCG AAGCTT CTATCCCTTATCGTCGTCATCCTTGTAGTC CCTGGCACTGTAGACCTCT**

pPN081

JPN239 **CGTAG CTGCAG ATGTATCAGTTAGTGAATG**

JPN127 **ATCCG AAGCTT CTATCCCTTATCGTCGTCATCCTTGTAGTC CCTGGCACTGTAGACCTCT**

pPN083

JPN261 **CCTTG AAGCTT CTTGCAGGGCAAGTCCC**

JPN262 **GCCAG CCATGG TTATTTATTCGCTGATT**
